# Scatter-Free UV–Visible Spectroscopy for Accurate and Precise RNA Quantification in Complex RNA Nanoparticle Formulations

**DOI:** 10.1021/acs.analchem.5c03644

**Published:** 2025-11-04

**Authors:** Aida López Espinar, Eric C. Le Ru, Parveen Kumar, Francisca Soares, Caitriona M. O’Driscoll, Brendan L. Darby, Piotr S. Kowalski

**Affiliations:** † School of Pharmacy, 8795 University College Cork, Cork T12 K8AF, Ireland; ‡ Marama Laboratories Limited, 32 Salamanca Road, Wellington 6012, New Zealand; § The MacDiarmid Institute for Advanced Materials and Nanotechnology, School of Chemical and Physical Sciences, 428627 Victoria University of Wellington, P.O. Box 600, Wellington 6140, New Zealand; ∥ Marama Laboratories Limited, DCU Alpha, Old Finglas Road, Glasnevin, Dublin D11KXN4, Ireland; ⊥ APC Microbiome Ireland, 8795 University College Cork, Cork T12 K8AF, Ireland

## Abstract

Ribonucleic acid (RNA)-based drugs showed the potential for treating wide range of diseases. Their successful clinical use depends on developing complex nanoparticle (NP) formulations made from diverse biomaterials. Accurately quantifying total RNA concentration in complex formulations is challenging, often requiring expensive, low-throughput methods or fluorescence-based assays like RiboGreen that rely on effective NP disruption. This study evaluates scatter-free absorption spectroscopy (SFAS), a UV/Visible method that removes light scattering from NP components and enables accurate total RNA quantification in intact NPs. To validate SFAS, we employed diverse RNA formulations, including lipid NPs, polymer and dendrimer hybrid lipid NPs, and cyclodextrin nanocomplexes, which exhibit physicochemical characteristics that can interfere with RNA quantification. Data obtained with SFAS were compared to fluorescent-based assays utilizing RiboGreen and SYTO 9 dyes, which bind to RNA in free or encapsulated forms, respectively. SFAS demonstrated superior accuracy, precision, and reproducibility than fluorescence-based methods across all formulations, particularly those showing resistance to disruption. RNA quantification by SFAS was less influenced by NP composition and measurement conditions, unlike the RiboGreen and SYTO 9. These findings demonstrate SFAS as a versatile and reliable alternative to fluorescence-based assays for accurate quantification of total RNA concentration in complex RNA NP formulations.

## Graphical abstract

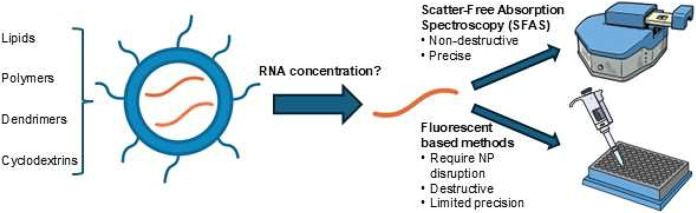

## Introduction

Ribonucleic acid (RNA) based drugs have emerged as a new class of biologics with the potential to revolutionize the treatment of a wide range of diseases.^1,2^ Clinical translation of RNA therapies requires a delivery system that is essential for protecting RNA from degradation and ensuring its safe and efficient intracellular delivery to target cells.^3^ Nonviral delivery systems, including nanoparticles (NPs) composed of lipids, polymers, dendrimers, and polysaccharides, are being actively investigated as RNA delivery vehicles in both preclinical and clinical settings.^4–8^ Importantly, the nature of the biomaterial plays a critical role in the NPs’ design, influencing nanoparticle characteristics such as ionization constant (p*K*_a_), surface charge distribution, lipophilicity, and RNA encapsulation efficacy, which impact their biocompatibility and intracellular RNA delivery capabilities.^9^

Improving the quality of preclinical research and facilitating the clinical translation of RNA NPs requires precise control over their formulation process and reliable characterization methodologies to ensure the reproducibility of the data. The formulation of NPs using microfluidic techniques and the characterization of their physicochemical properties, such as size and surface charge, have been well-established and extensively studied.^10–13^ However, accurately measuring RNA concentration in NP formulations remains challenging. For achieving an accurate and precise RNA quantification in NPs, scientists have to rely on a combination of highly complex, low-throughput, and costly techniques, including solvent extraction for RNA isolation, High-Performance Liquid Chromatography (HPLC), capillary electrophoresis (CE), and RT-dPCR, mainly applicable in the industry setting.^14–17^ While these techniques can also provide valuable information on RNA integrity and degradation kinetics, their broader application is often limited.^18^ On the other hand, fluorescence-based assays like RiboGreen are widely used for the assessment of both RNA concentration and encapsulation efficiency. However, this method suffers from poor precision, and its reliability depends on the efficient disruption of the NPs. Incomplete or inconsistent disruption, influenced by differences in NP composition, such as hydrophobicity, ionization capacity, and self-assembly, was shown to impact RNA quantification by RiboGreen, leading to inaccurate results.^19,20^ To address the above challenges in NP development, a universally applicable, straightforward, and reliable method for RNA quantification is essential.

UV/Visible (UV–vis) spectroscopy is a simple and widely used method for RNA quantification that relies on RNA absorption of UV light at a wavelength of 260 nm. However, its application for quantifying RNA encapsulated into NPs, with sizes typically ranging from 60 to 150 nm, is limited by the interference of light scattered by NPs. Scatter-Free Absorption Spectroscopy (SFAS) addresses this challenge by modifying the UV/vis spectroscopy method to eliminate scattered light interference and has demonstrated the capability to accurately and precisely measure RNA concentration in lipid nanoparticles.^21^ The SFAS method measures the absorption spectrum of RNA and other NP components while excluding the scattering effects from the NPs. This is achieved by placing the sample in a quartz cuvette at the center of an integrating sphere, a spherical cavity with highly reflective inner walls that trap and diffuse scattered light. While this setup effectively minimizes scattering interference, it affects the optical path length, requiring careful calibration to convert the transmission data into a true absorption spectrum independent of scattering.^21^ The nondestructive nature of SFAS also allows the reuse of the sample for further characterization and downstream nanoparticle analysis, improving sustainability. However, SFAS has so far only been evaluated in the context of LNPs containing ionizable lipids (SM-102), whereas many emerging nanoparticle formulations present distinct features from LNPs, including their chemical makeup, excipients composition, and particle size and morphology, which can interfere with UV-based RNA quantification by contributing to absorbance in the region around 260 nm. To address this, a linear deconvolution can be applied to the SFAS method to isolate the true RNA signal from background absorbance related to the nanoparticle components, thereby enabling accurate quantification.^21^

To further assess the applicability of SFAS for RNA quantification in NP research, we selected three representative classes of biomaterials, including polymers, dendrimers, and cyclodextrins, known for their efficient RNA delivery both in vitro and in vivo.^4,6,22^ To validate the robustness of SFAS method, we included materials that exhibit resistance to disruption by detergents such as Triton X-100, limiting the effectiveness of the RiboGreen assay, and possess physicochemical characteristics that could interfere with SFAS (e.g., aromatic groups). Specifically, we investigated a polymer–lipid hybrid NP (PH) containing an ionizable amino-polyester, and a cyclodextrin-based NP (CD) composed of amphiphilic cationic CDs complexed with RNA (Figure 1), both formulations showed resistance to disruption with detergents. Additionally, we included a dendrimer-lipid hybrid NP (DH) formulated with an amphiphilic Janus dendrimer featuring aromatic groups (Figure 1).^22–25^ The ionizable lipid SM-102 (LNP) formulation (Figure 1) was used as a control since it has been shown to exhibit minimal absorbance in the same region as the RNA with SFAS, and it represents a clinically relevant LNP formulation.^21,26^ With the selected panel of nanoparticle formulations, we aimed to rigorously assess the potential and limitations of SFAS against fluorescence-based methods. We compared it to the widely used RiboGreen assay and evaluated the ability of SYTO 9 cell-penetrating dye to address the reliance of current fluorescent-based methods on particle disruption.

**Figure 1:**
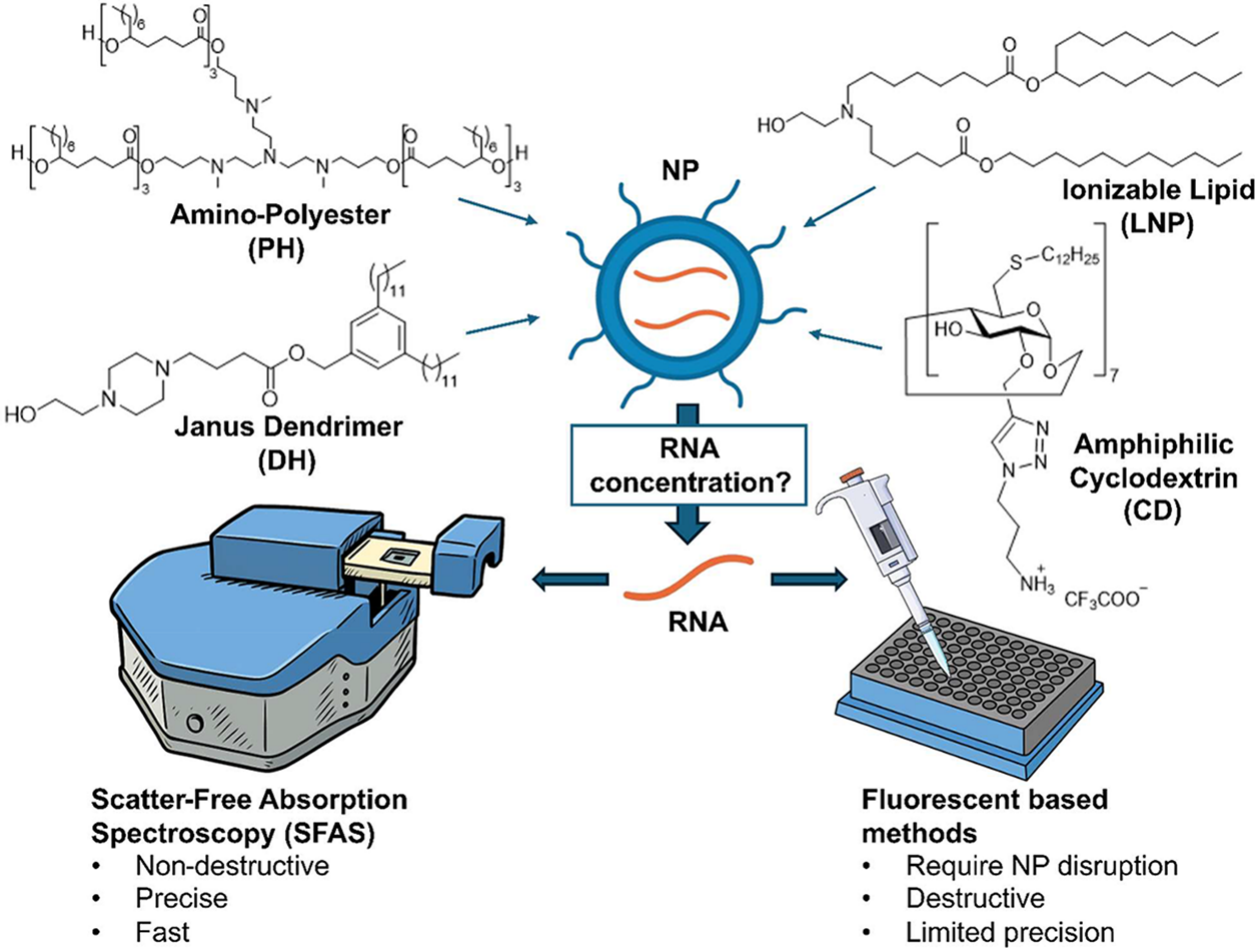
Schematic illustrating the study design, including selected materials and methodologies used to quantify total RNA concentration in NP formulations.

Our results demonstrate that SFAS enables accurate, precise, and reproducible RNA quantification in NP formulations that are incompatible with conventional fluorescence-based methods due to ineffective NP disruption. Notably, SFAS was effective even in systems with significant absorbance contribution in the RNA absorbance region around 260 nm. While SYTO 9 provided improved RNA detection compared to RiboGreen in hard-to-disrupt nanoparticles and produced values more consistent with those from SFAS, its broader applicability was limited. These findings underscore the robustness of SFAS across various complex nanocarrier compositions, supporting its broader application as a reliable tool for RNA quantification in nanoparticle research.

## Materials and Methods

### Formulation of Nanoparticles

Polymer–lipid (PH) and dendrimer-lipid hybrid nanoparticles (DH) were formulated with FLuc mRNA and EpCAM siRNA at a 1:3 ratio by microfluidics mixer using syringe pumps (Pump 33 DDS, Harvard Apparatus)^23^ or stirring ethanol and aqueous phases for 30 s at 1000 rpm using a microtiter plate stirrer MiXdrive 96 MTP with MIXcontrol MTP device (2MAG), respectively.

The ethanol phases were prepared by solubilizing a mixture of AA3-DD-3 or IAJD71, DOPE, cholesterol, and C14-PEG_2000_ at a molar ratio of 50:25:23.5:1.5. The aqueous phase contained FLuc mRNA or EpCAM siRNA in 10 mM citrate buffer pH 3.2.^23^ A Nitrogen to Phosphate (N/P) ratio of 9:1 of nitrogen in the polymer/dendrimer to phosphate in the RNAs was used for both formulations.

SM-102 lipid nanoparticles were formulated by mixing ethanol and aqueous phase at a 1:3 volumetric ratio in a microfluidic mixer, using syringe pumps.^23^ The ethanol phase contained a mixture of SM-102, DSPC, cholesterol, and DMG-PEG_2000_ at a molar ratio of 50:10:38.5:1.5. A N/P ratio of 6:1.^27^ The aqueous phase contained RNA in 10 mM citrate buffer pH 3.2.

Cyclodextrin nanoparticles (CD) were formulated by combining FLuc mRNA or EpCAM siRNA and the CD (1 mg/mL stock) at a 10:1 mass ratio in RNase-free water to achieve a 1000 nM final concentration of RNAs. The mixture was incubated in a ThermoMixer (Eppendorf) at 25 °C with shaking at 1000 rpm for 30 min.

### RNA Concentration Quantification with SFAS

To quantify RNA concentration in the nanoparticles using SFAS method, PH, DH, CD, and LNP samples were diluted to 5 μg/mL in PBS or RNase-free water. Dilutions were based on expected RNA content, considering possible losses from theoretical concentration. Empty NPs were prepared and diluted identically to RNA-loaded NPs.

All SFAS measurements were performed using a CloudSpec instrument (Marama Laboratories, Ireland). Measurements were performed using a standard 1 cm^2^ Quartz cuvette filled with 1 mL of solution. The cuvette was inserted inside an integrating sphere for SFAS measurement, and the instrument automatically corrected for path length modification effects in this geometry^28–30^ to report the absorption coefficient as equivalent optical density over 1 cm. Extinction spectra were measured at the same time in a standard transmission configuration, from which the scattering spectra were obtained by subtracting absorption from extinction. All spectra were measured against a water or PBS reference. For RNA and RNA-loaded NP samples, the buffer absorbance and extinction were also measured and subtracted from all spectra.

The RNA-loaded spectrum was fitted to a weighted sum of the pure RNA and empty NP reference spectra. The total RNA concentration was determined from the RNA spectrum weight, assuming the known concentration of the pure RNA reference sample. To determine the total RNA concentration in the starting solution, the dilution factors used for preparing the SFAS samples were applied: 4.6 for PH, DH, and LNP, and 3 for CD.

### Quantification of RNA Concentration with RiboGreen

The Quant-iT RiboGreen assay (ThermoFisher) was performed according to the manufacturer’s protocol with slight modifications. The assay was conducted in a flat-bottom black 96-well plate (Corning, Fisher Scientific). A standard curve was prepared by diluting RNA in TE 1× buffer to final concentrations ranging from 0 to 200ng/100μL. Each NP sample was diluted to a final volume of 100 μL in TE 1×. Then, 100 μL of RiboGreen (diluted 1:200 in TE 1×) was added to both the samples and the standard curve. The plate was incubated in the dark for 5 min, shaking at 100 rpm using an orbital shaker (Benchmark Scientific). After incubation, the fluorescence intensity of non-encapsulated RNA was measured using a Tecan SPARK plate reader (Tecan, Reading, UK) at an excitation wavelength of 485 (±20) nm and an emission wavelength of 535 (±25) nm. Next, 22 μL of 1% Triton X-100 was added to each sample, and the plate was incubated for 3 min, shaking at 100 rpm in the plate reader. Following incubation, the fluorescence intensity of total RNA was measured under the same conditions.

The RiboGreen assay protocol that includes heating was performed with the following modifications. The assay was performed in two separate black 96-well plates. Both plates contained a standard RNA curve and nanoparticle samples diluted in 1× TE buffer. In the first plate (nondisrupted condition), 100 μL of RiboGreen reagent (1:200 dilution in 1× TE) was added to each well, incubated in the dark for 5 min, shaking at 100 rpm at room temperature, and fluorescence was measured. In the second plate (disrupted condition), 22 μL of 1% Triton X-100 was added to each sample, followed by incubation at 95 °C for 20 min with shaking at 300 rpm using a ThermoMixer. After incubation, 100 μL of RiboGreen reagent (1:200 in 1× TE) was added to each well, incubated in the dark for 5 min, shaking at 100 rpm, and fluorescence was recorded under the same conditions.

RNA encapsulation efficiency (EE) was calculated as shown in eq 1. The total RNA concentration was calculated as shown in eq 2.

$$\%\mathrm{RNA}\;\mathrm{EE}=\frac{\left[\mathrm{Total}\;\mathrm{RNA}]-[\mathrm{Free}\;\mathrm{RNA}\right]}{\left[\mathrm{Total}\;\mathrm{RNA}\right]}\times 100$$ 1

Equation 1. RNA encapsulation efficacy

$$\mathrm{Total}\;\mathrm{RNA}\;\mathrm{concentration}=\frac{\mathrm{RNA}\;\mathrm{mass}\;\left(\mathrm{ng}\right)}{\mathrm{Sample}\;\mathrm{volume}}\times \mathrm{dilution}\;\mathrm{factor}$$ 2

Equation 2. Total RNA concentration

### Quantification of RNA Concentration with SYTO 9

RNA quantification was also performed using membrane-permeable SYTO 9 Green Fluorescent Dye (ThermoFisher) following the manufacturer’s instructions and a modified protocol adapted from RiboGreen assay. The assay was performed in a flat-bottom black 96-well plate. A standard curve was prepared by diluting RNA in PBS 1× to final concentrations ranging from 0 to 200ng/100μL. Each NP sample was diluted to a final volume of 100 μL in PBS 1×, subsequently, 50 μL of 2.5 μM SYTO 9 in PBS 1× was added to the samples and standard curve. The plate was incubated at room temperature in the dark for 30 min, with shaking at 100 rpm on an orbital shaker. Fluorescence intensity was measured using the plate reader at an excitation wavelength of 485 (±20) nm and an emission wavelength of 535 (±25) nm. The RNA concentration in the NP samples was determined by interpolating the fluorescence values against the standard curve. Using TE 1× as buffer, the assay was carried out following the same procedure as before.

## Results and Discussion

### Investigating SFAS for RNA Quantification in NPs with Diverse Composition

To evaluate the baseline absorbance of the NP components, we first measured the absorption spectra of the empty NPs (Figure 2A). The empty PH and CD NPs exhibited minimal (but not negligible) absorbance at 260 nm, very similar to the LNP control (Figure 2A, blue, orange, and purple lines). These findings indicate that the commonly used helper lipids, such as phospholipids (DOPE or DSPC), cholesterol, and PEGylated lipids (C14-PEG_2000_ or DMG-PEG), exhibit minimal absorbance at 260 nm. In contrast, the empty DH NPs exhibited strong absorbance across the 250–300 nm range (Figure 2A, green line), which could significantly impact RNA quantification by SFAS. The strong absorbance of the empty DH NPs is attributed to the chemical structure of IAJD71 compound, which contains a benzyl group (Figure 1).^22^ Such results highlight that materials containing resonance and aromatic structures, absorbing in the same wavelength region as RNA, can significantly affect RNA quantification. This observation aligns with the previously reported work,^21^ emphasizing the importance of considering the empty NP component absorbance for accurate RNA measurement by SFAS. Next, we measured the absorbance of the mRNA (Figure 2B–E, blue line) and siRNA-loaded NPs (Figure S1A–D blue line, Supporting Information). In both cases, the absorption spectrum of the RNA-loaded NPs closely resembles the free RNA, and it represents a linear combination of the absorption from both the empty NPs (Figure 2B–E, purple line, Figure S1A–D purple line, Supporting Information) and the free RNA-in-buffer (Figure 2B–E, green line, Figure S1A–D, green line, Supporting Information). Directly quantifying the RNA concentration using the 260 nm absorbance would lead to inaccuracies due to the previously described contributions from the NP components. Therefore, to achieve accurate quantification of the total RNA formulated into NPs, a linear deconvolution approach was used:^21^

$$A_{\mathrm{Loaded}\;\mathrm{NPs}}(\lambda )=\alpha A_{\mathrm{Empty}\;\mathrm{NPs}}(\lambda )+\beta A_{\mathrm{RNA}}(\lambda )$$

Where *A*_RNA_ represents the measured absorbance spectrum of the free RNA-in-buffer (*c*_0_ = 5 μg/mL). Meanwhile, *A*_Empty NPs_ corresponds to the scatter-free absorption of the empty NPs. The coefficients α and β represent the contribution of the empty NPs and free RNA-in buffers, respectively. After applying the linear deconvolution, we obtained a fitted absorbance spectrum for the RNA-loaded NPs (Figure 2B–E, orange line, Figure S1A–D, orange line, Supporting Information), which closely aligned with the original measured absorbance spectrum. This corrected spectrum effectively removes interference from the NPs components, ensuring more accurate RNA quantification. The RNA concentration can then be determined using the relation *c* = β*c*_0_.

**Figure 2:**
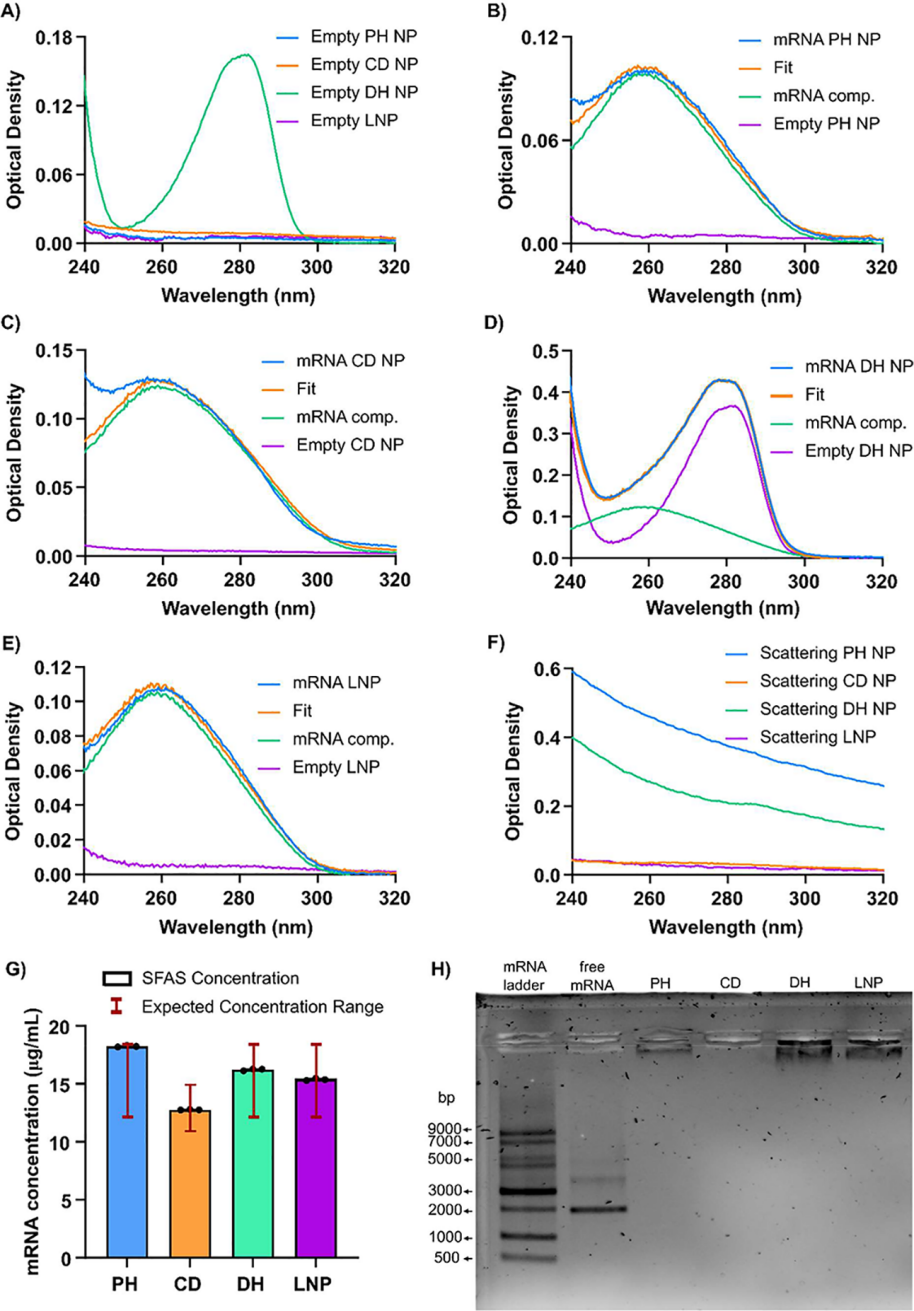
Impact of nanoparticle formulations on UV absorption and scattering for SFAS measurement. (A) Absorption spectrum of the empty NPs. The absorbance of loaded NPs (blue), fit (orange), mRNA (green), and empty NP (purple) for (B) PH, (C) CD, (D) DH, and (E) LNP. (F) Scattering spectrum of loaded NPs obtained using SFAS. (G) mRNA concentration measured with SFAS method after applying linear decomposition. Mean ± SD; *n* = 3, Technical replicates from a single independent NP batch are presented, demonstrating the precision of the method. (H) Agarose gel electrophoresis showing the NPs, free mRNA, and mRNA ladder.

The theoretical RNA concentrations for the PH, DH, and LNP formulations were calculated based on the initial RNA input and final volume, yielding a concentration of 23 μg/mL (Table 1). For the CD formulation, the RNA theoretical concentration was 15 μg/mL (Table 1). However, RNA losses during the preparation of PH, DH, and LNP nanoparticles, and dialysis are expected, leading to lower concentrations. In contrast, CD formulation does not involve purification or dialysis steps and is therefore considered technically lossless. Based on these considerations and our previous RNA quantification results using the RiboGreen assay,^25^ we expected RNA concentrations ranging from 13 to 18 μg/mL for PH, DH, and LNP formulations, and RNA concentrations of 12–15 μg/mL for the CD formulation (Table 1). To determine the total RNA concentration with SFAS, we applied the previously described linear deconvolution method and corrected for the dilution factors as described in the Materials and Methods section. By measuring RNA concentrations in triplicate for each nanoparticle batch (Figure 2G, Figure S1E, Supporting Information), we evaluate the precision of the method. In contrast, measuring RNA concentrations across three independent nanoparticle batches (Table 1) allows us to assess the reproducibility of the results, which fall within the expected concentration range while accounting for possible batch-to-batch differences arising from the formulation process. The RNA concentrations obtained for the NPs fell within the expected range (Table 1), demonstrating that the SFAS method is well suited for measuring RNA concentrations in NPs with diverse compositions and carrying different RNA cargos (mRNA/siRNA) without the need for particle disruption.

**Table 1:** Comparison of Total RNA Concentration Determined by SFAS Applying a Linear Deconvolution, RiboGreen, and SYTO 9^a^

formulation	RNA	theoretical concentration [μg/mL]	expected concentration [μg/mL]	SFAS total concentration [μg/mL]	RiboGreen total concentration [μg/mL]	SYTO 9 total concentration [μg/mL]
PH	mRNA	23	13–18	17.01 ± 1.18	8.79 ± 2.07^b^	11.88 ± 1.38^c^
CD		15	12–15	13.03 ± 0.40	1.35 ± 0.38^b^ ^d^	9.65 ± 0.63
DH		23	13–18	17.21 ± 1.59	16.81 ± 3.38	17.27 ± 1.94
LNP		23	13–18	13.38 ± 2.10	12.8 ± 0.92	15.02 ± 2.62
PH	siRNA	23	13–18	15.47 ± 0.48	8.14 ± 1.45^b^	9.86 ± 1.65^c^
CD		15	12–15	14.02 ± 1.43	1.47 ± 0.42^b^ ^d^	12.34 ± 0.85
DH		23	13–18	18.59 ± 2.18	15.81 ± 1.72	17.22 ± 1.69
LNP		23	13–18	16.00 ± 0.34	14.82 ± 2.59	16.90 ± 2.04

a The data in Table 1 represent mean ± SD from three independent NP batches (*n* = 3) assessing the reproducibility of the methods. Note that for SFAS, the SD is caused by the batch-to-batch variation and is much smaller for repeats of the same NP batch.

b Indicates statistical significance between SFAS and RiboGreen, *p* < 0.001.

c Indicates statistical significance between SFAS and SYTO 9, *p* < 0.05.

d Indicates statistical significance between RiboGreen and SYTO 9, *p* < 0.0001.

Additionally, these data highlight the accuracy and precision of the SFAS method for RNA quantification across a range of nanoparticle sizes, from 70 to 300 nm (Figure S2A, Supporting Information). While it is generally expected that nanoparticles with larger hydrodynamic diameters produce greater light scattering (per particle), the SFAS scattering spectrum is also proportional to particle concentration. NPs with a size of approximately 100 nm (PH and DH) (Figure S2A, Supporting Information) exhibited more pronounced scattering than the CD formulation, which has a larger diameter (Figure 2F).^31^ The lower particle concentration of the CD formulation compared to PH and DH, measured by Multiangle dynamic light scattering (Table S1, Supporting Information), could explain its lower scattering despite larger particle size. Despite these variations, the SFAS method effectively compensates for scattering artifacts, enabling reliable RNA quantification across all tested NPs. Furthermore, our findings support the use of deconvolution as an efficient method to remove the absorbance contributions in the range of 250–300 nm produced by the NP components.^21^

It should be noted that the SFAS method quantifies the total RNA present in the solution since the absorption at 260 nm cannot distinguish between free and encapsulated RNA. To confirm that the RNA concentrations measured by SFAS mainly represent encapsulated RNA, we performed gel electrophoresis for the mRNA (Figure 2H) and the siRNA formulations (Figure S1F, Supporting Information) to visualize the mobility of RNA not associated with the NPs. The gel electrophoresis of the NPs showed no detectable free RNA in the NP samples compared to the free RNA controls, confirming that the RNA quantified by SFAS is encapsulated in the NPs.

### Comparative Analysis of RNA Quantification Using SFAS and Fluorescence-Based Methods (RiboGreen and Syto9)

We evaluated SFAS against different fluorescence-based RNA quantification methods. The first included the widely used RiboGreen assay, based on a membrane-impermeable dye that fluoresces upon binding to RNA. However, accurate measurements with this method require nanoparticle disruption, typically using detergents or solvents, to make the encapsulated RNA accessible for binding to the dye. This disruption step depends on the nanoparticle’s composition, structural, and chemical properties of the ionizable cationic biomaterial, which can hinder RNA release and compromise quantification accuracy. The assay’s reliance on complete NP disruption is a major limitation, as any incomplete release of RNA leads to inaccurate results, particularly in those formulations resistant to detergent disruption.

To measure RNA concentrations in NPs, we performed the RiboGreen assay as outlined in the Materials and Methods section. The absence of fluorescence from empty nanoparticles and the lack of their impact on free mRNA concentration confirmed that the biomaterials did not interfere with the RiboGreen signal (Figure S3A, B, Supporting Information). We found that RiboGreen generally exhibited lower RNA concentrations compared to the SFAS method across all evaluated formulations (Table 1). A similar discrepancy between SFAS and RiboGreen was previously reported by Le Ru et al., who observed approximately a 15% difference in RNA quantification between the two methods.^21^ The RNA concentrations for DH and LNP differed by ∼3.5 and ∼4.5%, respectively, between SFAS and RiboGreen for both RNA cargos. Although the differences in measurement between those two methods were not significant, SFAS demonstrates greater accuracy as compared to our expected RNA concentrations (Table 1). In contrast, the RNA concentrations measured by RiboGreen and SFAS for PH and CD differed by ∼48 and ∼90%, respectively. In both cases, RiboGreen significantly underestimated the RNA concentrations compared to the expected values based on the formulation RNA input (Table 1). This significant underestimation is due to insufficient disruption of the nanoparticles with Triton X-100. This issue is particularly pronounced in formulations with high charge density, where protonated amines create strong electrostatic interactions with RNA, or in systems that lack lipid excipients, both of which increase resistance to disruption by detergents and make RNA quantification more difficult.^32^ For example, the amino polyester used to formulate polymer–lipid hybrid NPs (Figure 1) contains four tertiary amines,^23^ which likely enhance electrostatic interactions between the polymer and the mRNA, affecting the NP’s disruption and consequently accurate quantification by RiboGreen. Amphiphilic Cyclodextrins (Figure 1),^24^ which form complexes with RNA without lipid excipients, are similarly resistant to disruption under the same conditions as reflected by data in Table 1. The RiboGreen assay provides only an estimated RNA encapsulation for the CD formulations (Figure 3A, Figure S4A, Supporting Information), as the fluorescence signal remained low both before and after Triton addition due to ineffective nanoparticle disruption. While a low RiboGreen signal in the intact CD nanoparticles indicates the absence of free RNA, the RNA encapsulation in the CD formulations was further confirmed by gel electrophoresis (Figure 2H, Figure S1F, Supporting Information), which showed no detectable free RNA. Several studies have explored alternative approaches for improving NP disruption for RNA quantification using the RiboGreen assay. For instance, Tween 20 and other surfactants have been investigated as a substitute for Triton X-100.^33^ Additionally, the use of heparin, a negatively charged polysaccharide that competes with RNA for binding to positively charged NP components, has been reported.^34^ Despite these modifications, the accuracy and reliability of the RiboGreen assay for difficult-to-disrupt formulations remain challenging. To improve the NPs disruption, we modified RiboGreen protocol by introducing a heating step at 95 °C for 20 min with 1% Triton X-100. This high-temperature treatment was intended to improve disruption by accelerating detergent-driven fragmentation of the nanoparticle structure.^35^ This heating step led to a notable increase in RNA recovery only for the PH formulation containing mRNA (Figure 3B). For the siRNA formulations, heating resulted in a significant increase in RNA concentration across all formulations except for the CD formulation (Figure S4B, Supporting Information). While this modification slightly improves RNA release, it does not fully overcome the limitations of fluorescent assays for hard-to-disrupt nanoparticles.

**Figure 3:**
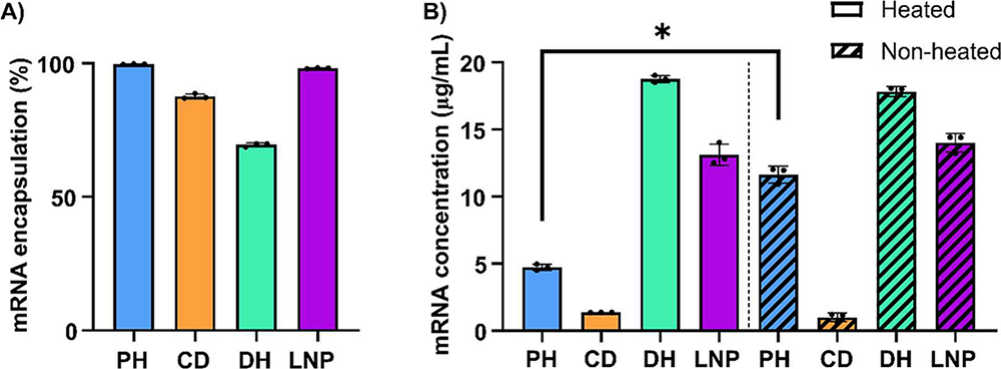
Encapsulation Efficiency (EE) and Impact of Heating on RNA Quantification by RiboGreen. (A) RiboGreen mRNA EE. (B) mRNA concentration for RiboGreen assay heated and at room temperature. All data are presented as mean ± SD of *n* = 3 (technical replicates).* *p* < 0.05. The EE for CD formulation is an estimation, as accurate quantification is not possible due to incomplete nanoparticle disruption.

In search of an alternative fluorescence-based approach that may not require particle disruption, we explored the use of membrane-permeable dyes as an alternative to RiboGreen. SYTO 9 is a cell-penetrant dye that can bind indiscriminately to both DNA and RNA, emitting a strong green fluorescent signal with a peak emission at 503 nm upon binding. Due to its ability to penetrate both live and dead cells, SYTO 9 has been primarily used as a live/dead viability assay tool.^36,37^ Given its capacity to penetrate cells and interact with nucleic acids, we hypothesized that it could also penetrate NPs and bind to the encapsulated RNA, enabling quantification without requiring NP disruption, similar to SFAS. We also confirmed that empty nanoparticles did not interact with SYTO 9, ensuring that the measured signals reflected dye-RNA interactions (Figure S3C, Supporting Information).

To optimize the SYTO 9 assay for RNA quantification in NPs, we evaluated different buffer conditions, including PBS 1× and TE 1×. We found that the buffer had a strong impact on the RNA concentration measurements with STYO 9. In TE 1×, SYTO 9 could successfully bind to the free RNA in the standard curve (Figure S5A, Supporting Information). However, when applied to NP samples, the RNA concentrations measured in TE 1× deviated considerably from our expected values and did not align with measurements obtained using either SFAS or RiboGreen (Figure 4A, Figure S6A, Supporting Information). This suggests that while TE 1× supports dye-RNA interaction in solution, it may hinder the dye’s access to the encapsulated RNA in the nanoparticles. Conversely, when PBS 1× was used as a buffer, SYTO 9 also showed effective binding to the free RNA in the standard curve, but the fluorescence signals were lower than those obtained in TE 1× (Figure S5B, Supporting Information). The RNA concentrations measured in NP formulations using SYTO 9 in PBS 1× were much more consistent with those obtained using SFAS and more accurate than those measured with RiboGreen, particularly for difficult-to-disrupt formulations (Table 1, Figure 4B, Figure S6B, Supporting Information). For example, RNA concentrations measured using SYTO 9 differed by 30% from SFAS for the PH formulation (Table 1). In contrast, differences between CD, DH, and LNP formulations were all below 20%, reflecting better agreement. When compared to RiboGreen, SYTO 9 measurements differed by less than 25% for PH, DH, and LNP, but showed a substantial 88% discrepancy for CD formulation. Unlike RiboGreen, which requires complete nanoparticle disruption for accurate RNA quantification, SYTO 9 used in PBS 1× was able to access and bind encapsulated RNA without disrupting the particles. Although RiboGreen and SYTO 9 did not show significant differences in most formulations (PH, DH, and LNP), RiboGreen values deviated more from SFAS measurements than those obtained with SYTO 9 (Table 1). These results demonstrate stronger alignments between SYTO 9 and SFAS than with RiboGreen, highlighting its potential as an alternative method of RNA quantification, particularly for challenging NP formulations.

**Figure 4:**
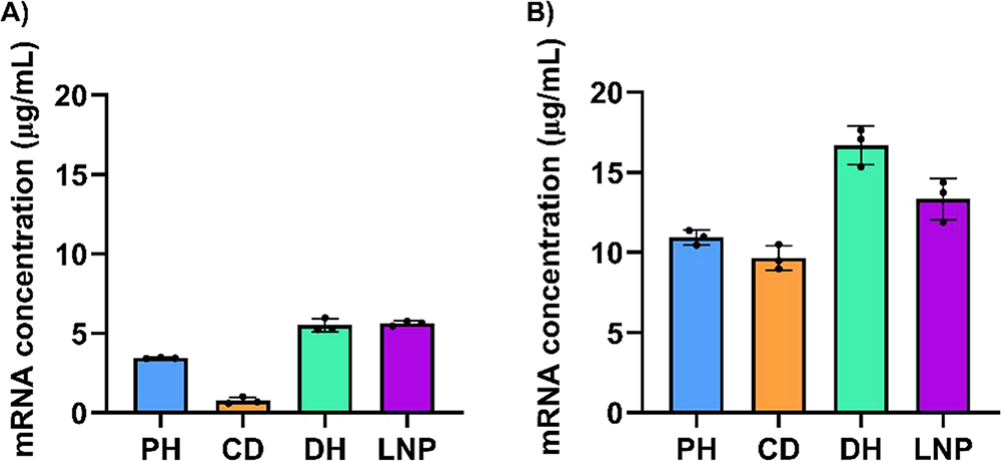
mRNA concentration determined with SYTO 9 (A) in TE 1× buffer, (B) in PBS 1× buffer. Data are presented as mean ± SD of *n* = 3 (technical replicates).

Although SYTO 9 showed potential for RNA quantification in hard-to-disrupt nanoparticles, its broader applicability is limited by high sensitivity to buffer composition, RNA type and sequence, and temperature. These factors compromise its reliability and highlight the need for further optimization, including exploring alternative SYTO dyes with improved nucleic acid binding properties.^38^

Another limitation of RiboGreen and SYTO 9 is their incompatibility with fluorescently labeled RNAs, due to spectral overlap with common fluorophores like FITC, Cy3, and FAM. This interference can distort fluorescence signals and hinder accurate quantification. In contrast, SFAS quantifies RNA via absorbance at 260 nm, avoiding this overlap and enabling reliable measurement even with fluorescent labels.

## Conclusions

In conclusion, this study demonstrates that SFAS is a versatile, cost-effective, and robust method that allows accurate, precise, and reproducible total RNA quantification across different nanoparticle formulations, including lipids, polymers, dendrimers, and cyclodextrins. SFAS shows particularly improved accuracy for delivery systems that often resist disruption by detergents, which limits the reliability of RNA measurement with fluorescent-based methods. By applying linear deconvolution, the SFAS method can effectively separate the absorbance contributions of nanoparticle components from those of RNA, allowing accurate quantification even in the presence of UV-absorbing biomaterials containing aromatic structures. Compared to fluorescence-based assays like RiboGreen and SYTO 9, SFAS consistently shows superior precision and better agreement with expected RNA concentration. While SYTO 9 offers some advantages over RiboGreen by avoiding the need for NP disruption, its sensitivity to buffer conditions, temperature, and RNA variability undermines its consistency. Overall, these findings highlight SFAS as a reliable and broadly applicable technique for RNA quantification in complex NP formulation, supporting the development of RNA-based nanomedicines.

## Supplementary Material



## Notes

Nanoparticles characterization (DLS, Z-Potential), standard curves for SYTO 9 assay, and polymer, dendrimer and mRNA synthesis and characterization (1H-NMR, GPC, ESI) (PDF)
