# Supplementary material for: Scatter-Free UV–Visible Spectroscopy for Accurate and Precise RNA Quantification in Complex RNA Nanoparticle Formulations
Source: Anal Chem. 2025 Nov 4;97(45):24928–35. doi: 10.1021/acs.analchem.5c03644 (PMC12631730; doi:10.1021/acs.analchem.5c03644)
Supplement: Supplementary file 1 [file ac5c03644_si_001.pdf]

Supporting Information

Scatter-Free UV-Visible Spectroscopy for Accurate and Precise RNA Quantification in Complex RNA Nanoparticle Formulations

Aida López Espinar[1], Eric C. Le Ru[2,3], Parveen Kumar[1], Francisca Soares[1], Caitriona M. O'Driscoll[1], Brendan L. Darby[4], Piotr S. Kowalski[1,5,]*

1. School of Pharmacy, University College Cork, Cork, T12 K8AF, Ireland
2. Marama Labs Limited, 32 Salamanca Road, Wellington, 6012, New Zealand
3. The MacDiarmid Institute for Advanced Materials and Nanotechnology, School of Chemical and Physical Sciences, Victoria University of Wellington, P.O. Box 600, Wellington 6140, New Zealand
4. Marama Labs Limited, DCU Alpha, Old Finglas Road, Glasnevin, Dublin, D11KXN4, Ireland
5. APC Microbiome Ireland, University College Cork, Cork, T12 K8AF, Ireland

*piotr.kowalski@ucc.ie

Table of contents:

- **Figure S1:** Impact of nanoparticle formulations on UV absorption and scattering for SFAS measurement.
- **Figure S2:** Nanoparticle characterization by DLS.
- **Table S1:** Nanoparticle concentration determined by MADLS
- **Figure S3:** mRNA concentration of empty nanoparticles.
- **Figure S4:** Encapsulation Efficiency and Impact of Heating on siRNA Quantification by RiboGreen.
- **Figure S5:** SYTO 9 assay standard curves.
- **Figure S6:** siRNA concentration determined with SYTO 9.
- **Materials**
- **Instrumentation and characterization**
- **Synthesis and Characterization AA3-DD-3 polymer**
- **Synthesis of 3,5-Bis(dodecyloxy)benzyl 4-(4-(2-hydroxyethyl)piperazin-1-yl)butanoate (DH)**
- **mRNA synthesis and characterization**
- **Hydrodynamic diameter and surface charge characterization**
- **Nanoparticle concentration determined by multi-angle DLS (MADLS)**
- **Gel electrophoresis analysis**
- **Statistical Analysis**

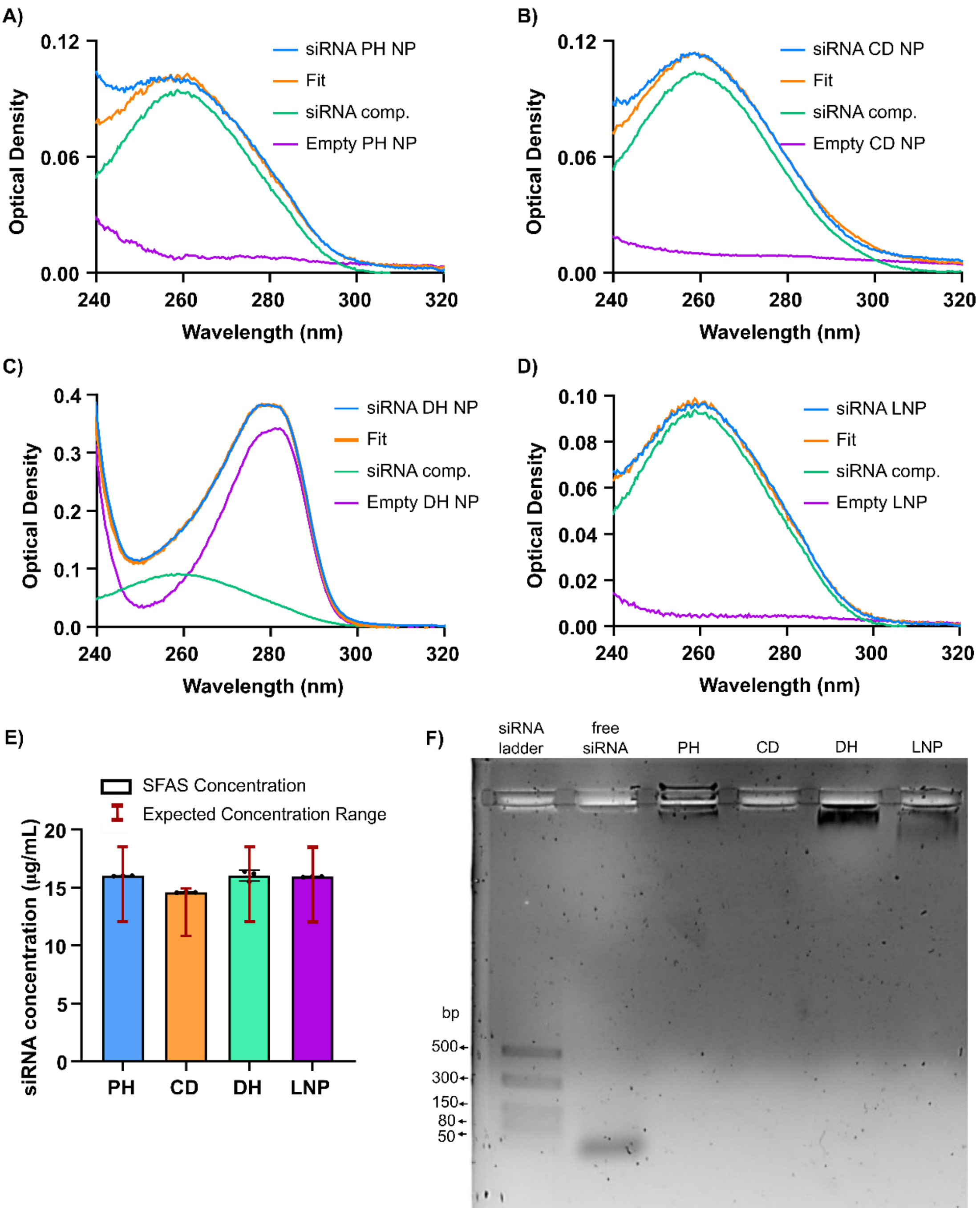

Figure S1. Impact of nanoparticle formulations on UV absorption and scattering for SFAS measurement. The absorbance of loaded NPs (blue), fit (orange), siRNA (green), and empty NP (purple) for A) PH, B) CD, C) DH, and D) LNP. E) siRNA concentration measured with SFAS method after applying linear decomposition. Mean ± SD; n = 3, Technical replicates from a single independent NP batch are presented, demonstrating the

precision of the method. F) Agarose gel electrophoresis showing the NPs, free siRNA, and siRNA ladder.

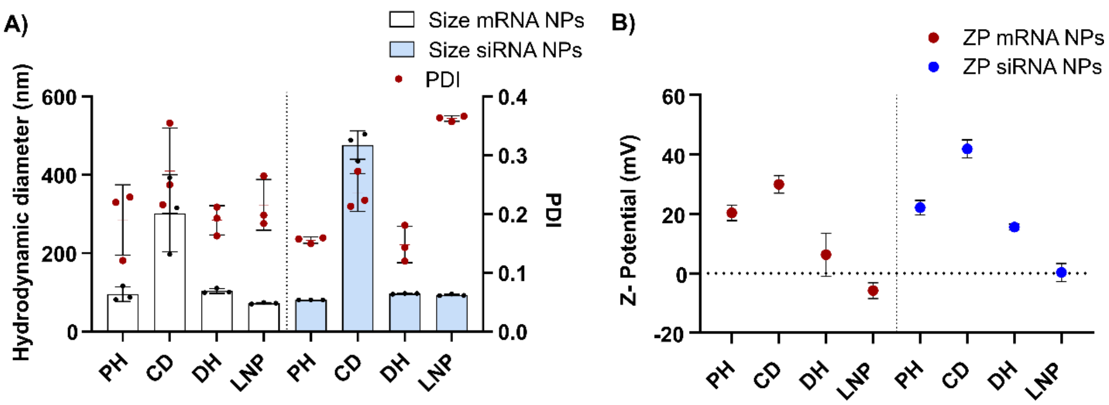

Figure S2. Nanoparticle characterization by DLS. A) Hydrodynamic diameter (nm) and polydispersity index (PDI) of the NPs. Data are presented as mean ± SD; n = 3 (independent batches). B) Surface charge of NPs. Data are presented as mean ± SD; n = 3 (independent batches).

Table S1. Nanoparticle concentration determined by MADLS

Formulation	Total number concentration (particles/mL)
CD	5.52E+07
PH	1.03E+10
DH	1.71E+10

CD, PH, and DH nanoparticle concentration (particles/mL) determined by multi-angle DLS (MADLS).

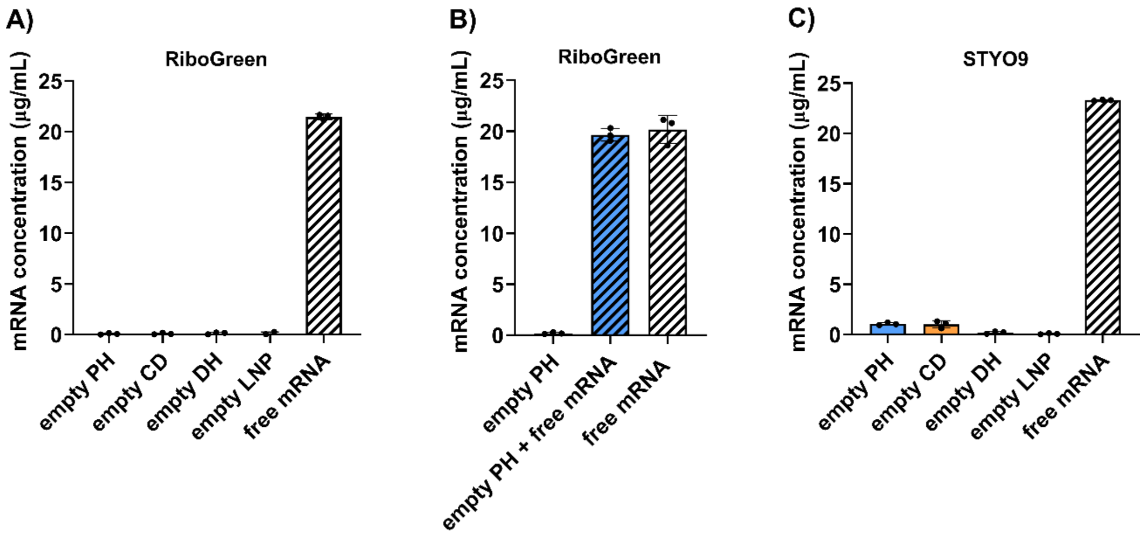

Figure S3. mRNA concentration of empty nanoparticles determined by A) RiboGreen, C) SYTO 9. Free mRNA in PBS buffer was included as a positive control. B) mRNA concentration of a mixture of empty PH NPs with free mRNA, showing that the empty NPs do not interact with free mRNA, allowing full mRNA quantification. Empty PH and free mRNA alone were included as controls.

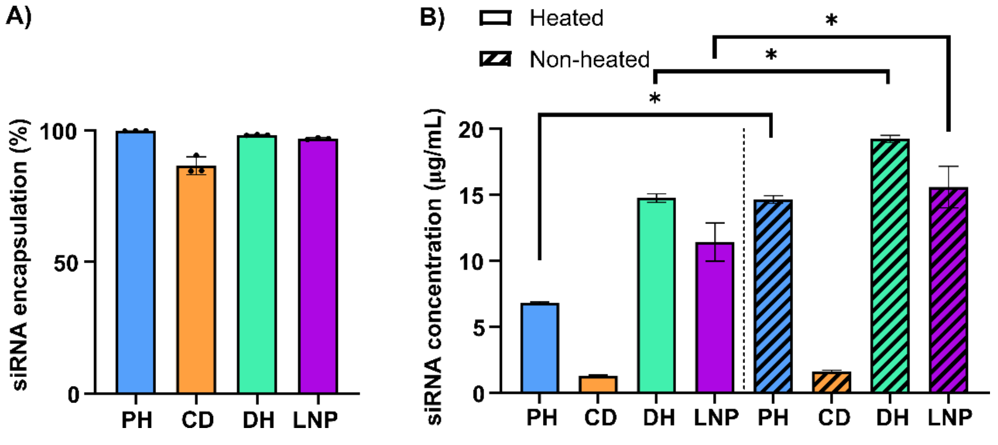

Figure S4. Encapsulation Efficiency and Impact of Heating on siRNA Quantification by RiboGreen. A) RiboGreen siRNA encapsulation efficacy, B) siRNA concentration for Ribogreen assay heated and at room temperature. All data are presented as mean ± SD of n = 3 (technical replicates).* $p < 0.05$

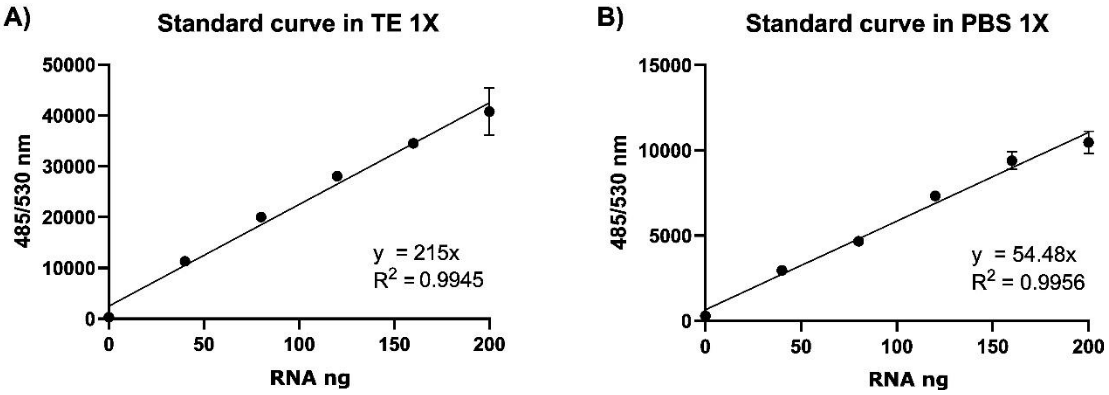

Figure S5. SYTO 9 assay standard curves. FLuc mRNA standard ranging from 0 to 200 ng A) in 1X TE buffer, B) in 1X PBS buffer.

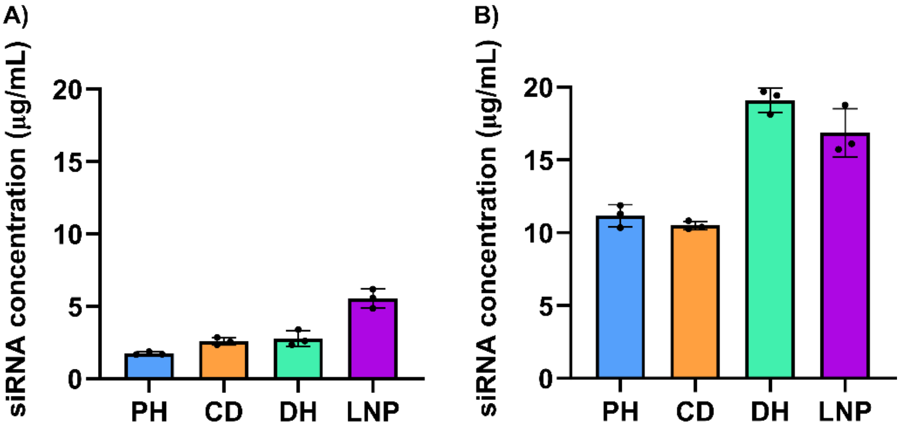

Figure S6. siRNA concentration determined with SYTO 9 A) in TE 1X buffer, B) in PBS 1X buffer. Data are presented as mean ± SD of n = 3 (technical replicates).

Materials

The lipids 1,2-dioleoyl-sn-glycero-3-phosphoethanolamine (DOPE), 1,2-dioctadecanoyl-sn-glycero-3-phosphocholine (DSPC), 1,2-Dimyristoyl-rac-glycero-3-methoxypolyethylene glycol-2000 (DMG-PEG2000) and 1,2-dimyristoyl-sn-glycero-3-phosphoethanolamine-N-[methoxy-(polyethyleneglycol)-2000](ammonium salt) (C14-PEG_{2000}) and the sterol cholesterol were purchased from Avanti Research. The AA3-DD-3 amino polyester [1], the Janus-type dendrimer (IAJD71)[2] and the amphiphilic cationic cyclodextrin (CD)[3] were synthesized and characterized as previously described

(Synthesis of materials, Supplementary Information). The lipid SM-102 was purchased from Tebubio. EpCAM siRNA was purchased from Horizon Discovery (J-004568-11-0050).

Instrumentation and characterization

Molecular weight and polydispersity (Đ) of the polymers were determined by Gel Permeation Chromatography (GPC) using a Tosoh EcoSEC HLC-8320GPC with refractive index (RI) detector, carried out in tetrahydrofuran (THF) mobile phase calibrated with linear polystyrene standards on TKSgel G3000+4000HHR column operating at 1.0 mL min-1. Samples were filtered through 0.45 μm PTFE filters (Fisher Scientific Ireland) before injections and at approximately 2 mg mL-1 polymer concentration.

^{1}H-NMR spectra were recorded on a Bruker 400 MHz NMR spectrometer in deuterated chloroform (CDCl3, Sigma Aldrich Ireland). The chemical shifts were referenced to the residual proton resonance of the solvent peak at 7.26 ppm. ^{13}C-NMR spectra were recorded on a Bruker 400 MHz NMR spectrometer in deuterated chloroform (CDCl3). The chemical shifts were referenced to the residual proton resonance of the solvent peak at 77.160 ppm. Chemical shifts (δ) are reported in parts per million (ppm). Splitting patterns are reported as follows: singlet (s), doublet (d), triplet (t), quadruplet (q), quintuplet (quint), multiplet (m). All NMR spectra were processed using MestReNova NMR software, version 12.0.0-20080 (Mestrelab Research S.L.).

Nominal mass spectra were recorded on a Waters Quattro Micro triple quadrupole instrument in electrospray ionization (ESI) mode using 50% acetonitrile-water containing 0.1% formic acid as eluent; samples were prepared at a concentration of approximately 1 mg mL-1 in water.

Synthesis and Characterization AA3-DD-3 polymer

Synthesis of AA3-DD-3 polymer (PH)

AA3-DD-3 was synthesized via ring opening polymerization (ROP) of the lactone δ-Dodecalactone (DD) in the presence of an amino-alcohol 3,3',3''-((nitrilotris(ethane-2,1-diyl))tris (methylazanediyl))tris(propan-1-ol) (AA3) as initiator and TBD as catalyst in bulk at room temperature. The monomer to the initiator hydroxyl group ratio was set equal to 3 in order to obtain APEs with 3 units of lactones for each arm. The hydroxyl group of the initiator to the catalyst molar ratio was set equal to 6.3. 9 equivalents of DD, 1 equivalent of AA3, and 24 mg of Na2SO4 anhydrous were poured into a 10 mL vial and left to stir for 15 min. Then the mixture was poured into another vial with 47 mg of TBD and 24 mg of Na2SO4 anhydrous and was left to react under vigorous stirring for 24 h at room temperature. The polymerization was stopped by adding an excess of benzoic acid in dichloromethane or diethyl ether (1 mmol mL-1). The final mixture was further diluted in dichloromethane and washed three times with a saturated solution of NaCl. The organic phase was recovered, dried with Na2SO4 anhydrous filtered with PTFE 0.45 μm filter, and the solvent was removed under reduced pressure to obtain the purified AA3-DD-3 as a viscous syrup. The final product was characterized via GPC and 1H-NMR (CDCl3, Bruker, 400 MHz) before and after purification.

Amino-alcohol AA3: 1H NMR (400 MHz, CDCl3) δ 3.75 (t, 6H, J = 4 Hz, H1), 2.62-2.54 (m, 12H, H5, H6), 2.48 (t, 6H, J = 8 Hz, H3), 2.25 (s, 9H, H4), 1.67 (quin, 6H, J = 4, 8 Hz, H2). 13C NMR (MHz, CDCl3) δ 63.66 (C1), 57.60 (C5), 55.60 (C3), 52.63 (C6), 42.66 (C4), 28.20 (C2). ESI C18H42N4O3 (M + H+) calcd. 362.6 obsd. 363.3.

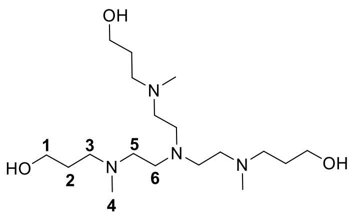

Polymer AA3-DD-3

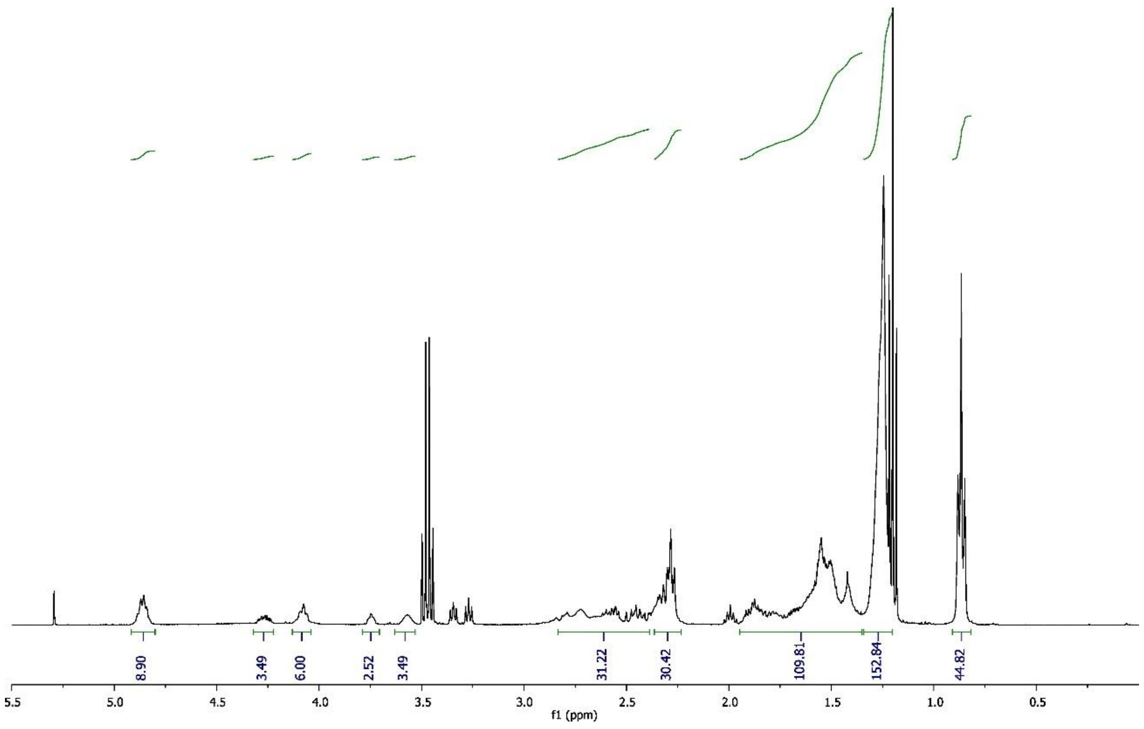

Figure S7. ^{1}H-NMR for AA3-DD-3.

Table S2. ^{1}H-NMR Characterization of the polymer AA3-DD-3.

APE	Mn_{theo}	Conversion (%)	q_{NMR}
AA3-DD-3	2147.26	78	2.1

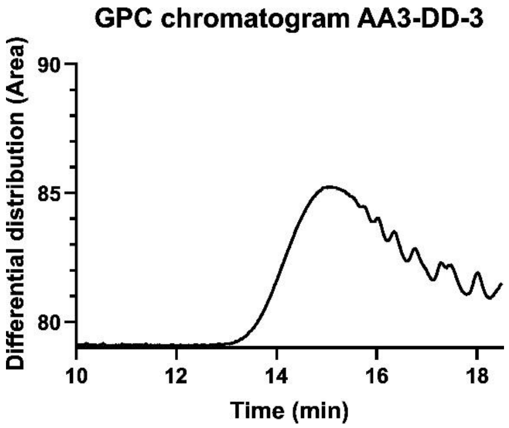

Figure S8. GPC chromatograms for AA3-DD-3.

Table S3. Characterization of AA3-DD-3 by Gel Permeation Chromatography (GPC).

[illegible]	[illegible]	[illegible]
[illegible]	[illegible]	[illegible]

Data are represented as mean ± SD from two independently synthesized batches of polymer. Mn: number average molecular weight. Đ: dispersity.

Synthesis of 3,5-Bis(dodecyloxy)benzyl 4-(4-(2-hydroxyethyl)piperazin-1-yl)butanoate (DH)

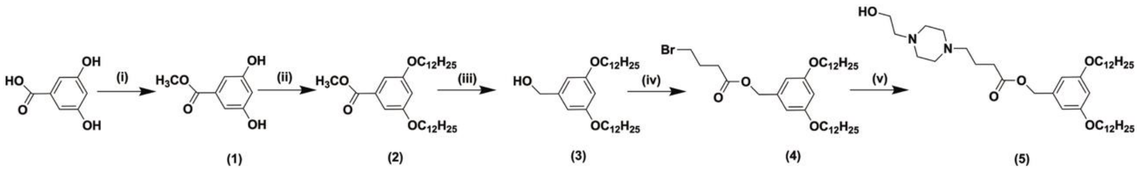

Reagents and conditions: (i) H_2SO_4 (cat.), MeOH, reflux, 2 h; (ii) $C_{12}H_{25}Br$, K_2CO_3, DMF, 120 °C, 2 h; (iii) $LiAlH_4$, THF, 0–23 °C, 1 h; (iv) 4-bromobutyric acid, EDC.HCl, DMAP, DCM, 0–23 °C, 12 h; (v) 1-(2-hydroxyethyl)piperazine, K_2CO_3, ACN, 95 °C, 3 h.

3,5-Dihydroxybenzoic acid (5g, 32.44 mmol) was dissolved in 50 mL MeOH. 0.5 mL conc. H_2SO_4 was added and the mixture was refluxed at 80 °C. Upon completion of the reaction, the solvent was removed under reduced pressure. The crude solid was treated

with saturated $NaHCO_3$ solution, and the resulting mixture was extracted with ethyl acetate. The combined organic layers were dried over anhydrous Na_2SO_4, filtered, and concentrated to yield a white solid **(1)** (Yield: 5.1 g, 93.5%).

Methyl 3,5-dihydroxybenzoate **(1)** (4 g, 23.79 mmol) and K_2CO_3 (9.87 g, 71.37 mmol) were suspended in dry DMF. 1-bromooctane (13.64 g, 54.71 mmol) was added to it and the reaction mixture was heated at 120 °C under a nitrogen atmosphere for 3 h. After completion, the mixture was cooled, poured into ice-cold water, and the precipitate was collected by filtration. The solid was dissolved in DCM, washed with water, dried over anhydrous Na_2SO_4, and concentrated to yield a white solid **(2)** (Yield: 11.4 g, 95%).

Methyl 3,5-dihydroxybenzoate **(2)** (5 g, 9.905 mmol) was dissolved in dry THF and added dropwise to a slurry of $LiAlH_4$ in THF (5.68 mL, 2M in THF) at 0 °C under nitrogen, and the mixture was stirred at RT for 1 h. The reaction was carefully quenched by the addition of water at 0 °C. The precipitated solid was filtered off, and the filtrate was dried over anhydrous Na_2SO_4, filtered, and concentrated to yield a light-yellow oil **(3)** (Yield: 3.98 g, 84.3%).

3,5-Bis(dodecyloxy)phenyl)methanol **(3)** (3.76 g, 7.88 mmol), 4-bromobutyric acid (1.32 g, 7.88 mmol), DMAP (0.286 g, 2.365 mmol), and EDC·HCl (1.51 g, 7.88 mmol) were dissolved in dry DCM and stirred at RT for 12 h. After completion, the mixture was washed with brine and extracted with DCM (3×). The combined organic layers were dried over anhydrous Na_2SO_4, filtered, and concentrated. Purification by flash column chromatography (hexanes/EtOAc gradient) yielded a colorless oil **(4)** (Yield: 3.7 g, 75%).

3,5-bis(dodecyloxy)benzyl 4-bromobutanoate **(4)** (2.5 g, 3.995 mmol, 1 equiv), 1-(2-hydroxyethyl)piperazine (0.5461 g, 4.19 mmol, 1.05 equiv), and K_2CO_3 (0.5797 mg, 4.19 mmol, 1.05 equiv) were dissolved in ACN (40 mL) and the mixture was heated at reflux

(95 °C) for 3 h. After completion, the reaction mixture was cooled to RT and ACN was removed under reduced pressure. Water (15 mL) was added and extracted by DCM (25 mL × 3). All the organic phases were collected, dried over anhydrous Na_2SO_4, filtered, and solvent was evaporated to get the crude product, which was purified by flash column chromatography (DCM/MeOH) to get the 3,5-bis(dodecyloxy)benzyl 4-(4-(2-hydroxyethyl)piperazin-1-yl)butanoate as a colorless oil **(5)** (2 g, 74%). **^{1}H NMR** (400 MHz, $CDCl_3$): δ 6.46 (d, 2H, Ph*H*), 6.39 (t, 1H, Ph*H*), 5.03 (s, 2H, -CH_2Ph), 3.92 (t, 4H, 2×PhOCH_2-), 3.60 (t, 2H, -CH_2CH_2OH), 2.56 (br, 1H, -CH_2CH_2O*H*), 2.50–2.36 (m, 14H, N(CH_2CH_2)$_2$, -OCOCH_2CH_2CH_2- and -CH_2CH_2OH), 1.82 (m, 2H, -OCOCH_2CH_2CH_2-), 1.75 (m, 4H, PhOCH_2C$H_2(CH_2)_9CH_3$), 1.85 (m, 4H, PhOCH_2CH_2C$H_2(CH_2)_8CH_3$), 1.28 (br, 32H, PhOC$H_2CH_2CH_2$(CH_2)$_8CH_3$), 0.90 (t, 6H, PhO(CH_2)$_{11}$CH_3). **^{13}C NMR** (101 MHz, $CDCl_3$): δ 173.1, 160.5, 138.0, 106.5, 100.9, 68.1, 66.2, 59.7, 57.3, 57.2, 52.7, 52.0, 32.1, 31.9, 29.7, 29.6, 29.6, 29.6, 29.4, 29.3, 29.3, 26.1, 22.7, 21.8, 14.1. Purity by HPLC: 98.5%. HRMS m/z of $[M + H]^+$ calculated for $C_{41}H_{75}N_2O_5$: 675.5671; Found: 675.5674.

mRNA synthesis and characterization

FLuc mRNA was synthesized as previously reported.[4] DNA plasmids containing a T7 promoter upstream of the sequence encoding for Firefly luciferase (FLuc) were used as templates for mRNA synthesis. DNA plasmids were linearized using restriction enzymes XbaI (New England Biolabs, Ipswich, MA) and transcribed using the HiScribe T7 RNA Synthesis Kit (New England Biolabs). To synthesize nucleoside-modified mRNA, uridine triphosphate was replaced with N1-methylpseudouridine (m1Ψ) triphosphate (TriLink, San Diego, CA) in the transcription reaction. mRNA was post-transcriptionally capped with Vaccinia Capping System (New England Biolabs) and mRNA Cap 2'-O-Methyltransferase (New England Biolabs), resulting in Cap1 structure. A poly(A) tail of approximately 120 nucleotides was added using *E.coli* Poly(A) Polymerase (New

England Biolabs). mRNA was purified using the Monarch RNA Cleanup Kit (New England Biolabs). RNA concentration was determined using a NanoDrop One (Thermo Scientific). Finally, purified mRNA contained a 5’ cap (Cap1), a 5’ and 3’ UTR derived from the human hemoglobin subunit beta (HBB) gene, a coding region as listed below, and a poly(A)tail. mRNA integrity and purity were characterized by agarose gel electrophoresis under denaturing conditions (Egel 2% EX gels, ThermoFisher). Gels were imaged using an iBright Imaging System (ThermoFisher). FLuc sequence is provided in the Supporting Information.

FLuc:

ATGGAAGATGCCAAAAACATTAAGAAGGGCCCAGCGCCATTCTACCCACTC
GAAGACGGGACCGCCGGCGAGCAGCTGCACAAAGCCATGAAGCGCTACGC
CCTGGTGCCCGGCACCATCGCCTTTACCGACGCACATATCGAGGTGGACATT
ACCTACGCCGAGTACTTCGAGATGAGCGTTCGGCTGGCAGAAGCTATGAAG
CGCTATGGGCTGAATACAAACCATCGGATCGTGGTGTGCAGCGAGAATAGC
TTGCAGTTCTTCATGCCCGTGTTGGGTGCCCTGTTCATCGGTGTGGCTGTGG
CCCCAGCTAACGACATCTACAACGAGCGCGAGCTGCTGAACAGCATGGGCA
TCAGCCAGCCCACCGTCGTATTCGTGAGCAAGAAAGGGCTGCAAAAGATCC
TCAACGTGCAAAAGAAGCTACCGATCATACAAAAGATCATCATCATGGATA
GCAAGACCGACTACCAGGGCTTCCAAAGCATGTACACCTTCGTGACTTCCC
ATTTGCCACCCGGCTTCAACGAGTACGACTTCGTGCCCGAGAGCTTCGACCG
GGACAAAACCATCGCCCTGATCATGAACAGTAGTGGCAGTACCGGATTGCC
CAAGGGCGTAGCCCTACCGCACCGCACCGCTTGTGTCCGATTCAGTCATGCC
CGCGACCCCATCTTCGGCAACCAGATCATCCCCGACACCGCTATCCTCAGCG
TGGTGCCATTTCACCACGGCTTCGGCATGTTCACCACGCTGGGCTACTTGAT
CTGCGGCTTTCGGGTCGTGCTCATGTACCGCTTCGAGGAGGAGCTATTCTTG

CGCAGCTTGCAAGACTATAAGATTCAATCTGCCCTGCTGGTGCCCACACTAT
TTAGCTTCTTCGCTAAGAGCACTCTCATCGACAAGTACGACCTAAGCAACTT
GCACGAGATCGCCAGCGGCGGGGCGCCGCTCAGCAAGGAGGTAGGTGAGG
CCGTGGCCAAACGCTTCCACCTACCAGGCATCCGCCAGGGCTACGGCCTGA
CAGAAACAACCAGCGCCATTCTGATCACCCCCGAAGGGGACGACAAGCCTG
GCGCAGTAGGCAAGGTGGTGCCCTTCTTCGAGGCTAAGGTGGTGGACTTGG
ACACCGGTAAGACACTGGGTGTGAACCAGCGCGGCGAGCTGTGCGTCCGTG
GCCCCATGATCATGAGCGGCTACGTTAACAACCCCGAGGCTACAAACGCTC
TCATCGACAAGGACGGCTGGCTGCACAGCGGCGACATCGCCTACTGGGACG
AGGACGAGCACTTCTTCATCGTGGACCGGCTGAAGTCCCTGATCAAATACA
AGGGCTACCAGGTAGCCCCAGCCGAACTGGAGAGCATCCTGCTGCAACACC
CCAACATCTTCGACGCCGGGGTCGCCGGCCTGCCCGACGACGATGCCGGCG
AGCTGCCCGCCGCAGTCGTCGTGCTGGAACACGGTAAAACCATGACCGAGA
AGGAGATCGTGGACTATGTGGCCAGCCAGGTTACAACCGCCAAGAAGCTGC
GCGGTGGTGTTGTGTTCGTGGACGAGGTGCCTAAAGGACTGACCGGCAAGT
TGGACGCCCGCAAGATCCGCGAGATTCTCATTAAGGCCAAGAAGGGCGGCA
AGATCGCCGTGTAA

Hydrodynamic diameter and surface charge characterization

Nanoparticle size, polydispersity index (PDI), and ζ-potential (ZP) were analyzed using dynamic light scattering (DLS) with a Zetasizer Ultra (Malvern Instruments, Worcestershire, UK) at 25°C. To determine the hydrodynamic diameter and PDI, samples were prepared by diluting 30 μL of NPs in 450 μL of PBS. For ζ-potential analysis, 15 μL of NPs were diluted in 750 μL of Milli-Q water to provide a suitable ionic environment for surface charge evaluation. Measurements were conducted using Polystyrene Latex as the reference material and water as the dispersant. Each parameter was measured in

triplicate for every sample to ensure accuracy and reproducibility. The hydrodynamic diameter was determined based on the Z-average measurements, while the PDI describes homogeneity of the particle size distribution in the formulation. The ζ-potential was measured to assess the surface charge. The reported values represent the average of three independent measurements, with standard deviations indicating variability between nanoparticle batches.

Nanoparticle concentration determined by multi-angle DLS (MADLS)

Nanoparticle concentration (particles/mL) was determined using dynamic light scattering with multi-angle capability (MADLS) (Malvern Instruments, Worcestershire, UK) in a 1 cm^2 disposable Polystyrol/Polystyrene cuvette. Before measuring the nanoparticle concentration, the size of the buffer was determined to obtain the Dispersant Scattering Mean Count Rate (kcps), a parameter needed to determine the particles/mL. Samples were prepared by diluting 60 µL of NPs in 940 µL of buffer (PBS for PH and DH, and water for CD). Particle concentration measurements are achieved by recording the time-averaged photon count rate scattered by the sample to perform a transformation of the size distribution to derive the absolute number of particles present. Measurements were performed at 25°C with three runs per sample following a 60s equilibration. Results were reported as the mean of three independent measurements.

Gel electrophoresis analysis

The RNA encapsulation of the NPs was investigated using a 1% Agarose gel in Tris-acetate-EDTA (TAE 1X) buffer. The nucleic acid staining safe view (NBS Biologicals, Huntingdon, UK) was added to the running buffer (TAE 1X) (6 µL/ 100 mL). For reference, a mRNA ladder (NEB), siRNA ladder (NEB), free Fluc mRNA, and free EpCAM siRNA were included as controls. The 300 ng of RNA per sample was loaded on the gel in a total of 30 µL containing 3 µL of BlueJuice Gel Loading Buffer (NEB).

The gel was run at 120 V for 40 min (Fisherbrand™ multiSUB™ Midi Horizontal Gel System in combination with the BioRad PowerPac Basic Power Supply) and imaged in the iBright Imaging System (ThermoFisher).

Statistical Analysis

All data were analyzed using GraphPad Prism 9 (La Jolla, CA, USA) and are presented as mean ± SD. Statistical analysis was conducted using one-way ANOVA followed by Tukey's or Dunnett's multiple comparisons test for comparing multiple replicate means, or the unpaired two-tailed Student's t-test (assuming equal variances) for comparing two replicate means. A p-value ≤ 0.05 was considered statistically significant.

References

(1) Kowalski, P. S.; Capasso Palmiero, U.; Huang, Y.; Rudra, A.; Langer, R.; Anderson, D. G. Ionizable Amino-Polyesters Synthesized via Ring Opening Polymerization of Tertiary Amino-Alcohols for Tissue Selective mRNA Delivery. *Adv. Mater.* **2018**, *30 (34)*, e1801151.

(2) Zhang, D.; Atochina-Vasserman, E. N.; Maurya, D. S.; Liu, M.; Xiao, Q.; Lu, J.; Lauri, G.; Ona, N.; Reagan, E. K.; Ni, H.; Weissman, D.; Percec, V. Targeted Delivery of mRNA with One-Component Ionizable Amphiphilic Janus Dendrimers. *J. Am. Chem. Soc.* **2021**, *143 (43)*, 17975–17982.

(3) O'Mahony, A. M.; Ogier, J.; Desgranges, S.; Cryan, J. F.; Darcy, R.; O'Driscoll, C. M. A Click Chemistry Route to 2-Functionalised PEGylated and Cationic β-Cyclodextrins: Co-Formulation Opportunities for siRNA Delivery. Org. Biomol. Chem. **2012**, *10 (25)*, 4954–4960.

(4) López Espinar, A.; Mulder, L. M.; Elkhashab, M.; Khan, Z.; Czarnocki-Cieciura, M.; Aburto, M. R.; Vucen, S.; Kowalski, P. S. Tailoring Alkyl Side Chains of Ionizable

Amino-Polyesters for Enhanced In Vivo mRNA Delivery. *ACS. Appl. Bio. Mater.* **2025**, *8,* 3958-3971.
